# Isolation and thermal stabilization of mouse ferroportin

**DOI:** 10.1002/2211-5463.13039

**Published:** 2020-12-17

**Authors:** Chandrika N. Deshpande, Corbin R. Azucenas, Bo Qiao, Norimichi Nomura, Vicky Xin, Josep Font, So Iwata, Tomas Ganz, Elizabeta Nemeth, Bryan Mackenzie, Mika Jormakka

**Affiliations:** ^1^ Structural Biology Program Centenary Institute Sydney NSW Australia; ^2^ Department of Pharmacology & Systems Physiology University of Cincinnati College of Medicine OH; ^3^ Medical Sciences Baccalaureate Program University of Cincinnati College of Medicine OH USA; ^4^ Department of Medicine David Geffen School of Medicine University of California Los Angeles CA USA; ^5^ Department of Cell Biology Graduate School of Medicine Kyoto University Japan; ^6^ Department of Pathology David Geffen School of Medicine University of California Los Angeles CA USA; ^7^ Present address: Transporter Biology Group Discipline of Pharmacology Sydney Medical School University of Sydney NSW 2006 Australia

**Keywords:** ferroportin, hepcidin regulation, iron metabolism, thermostabilization

## Abstract

Ferroportin (Fpn) is an essential mammalian iron transporter that is negatively regulated by the hormone hepcidin. Our current molecular understanding of Fpn‐mediated iron efflux and regulation is limited due to a lack of biochemical, biophysical and high‐resolution structural studies. A critical step towards understanding the transport mechanism of Fpn is to obtain sufficient quantities of pure and stable protein for downstream studies. As such, we detail here an expression and purification protocol for mouse Fpn yielding milligram quantities of pure protein. We have generated deletion constructs exhibiting enhanced thermal stability and which retained iron‐transport activity and hepcidin responsiveness, providing a platform for further biophysical studies of Fpn.

## Graphical abstract

Here, Deshpande *et al*. describe the production and purification of mouse ferroportin (Fpn), the only known mammalian iron efflux protein, and selective loop deletion constructs with enhanced thermal stability. Transport activity and hepcidin responsiveness of the constructs were assessed in *Xenopus* oocytes. The present work establishes a new protocol for the generation of stable Fpn protein in solution, constituting a platform for continued biophysical studies.

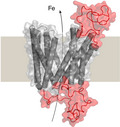

**Abbreviations**
ANOVA: analysis of variance
CPM: N‐[4‐(7‐diethylamino‐4‐methyl‐3‐coumarinyl)phenyl]maleimide
DDM: *n*‐dodecyl‐β‐d‐maltoside
Fpn: ferroportin
ICL: intracellular loop
MFS: major facilitator superfamily
Mw: molecular weight
SEC: size exclusion chromatography
Sf: *Spodoptera frugiperda*
*T*_m_: melting temperature
TM: transmembrane

Ferroportin (Fpn; SLC40A1) is the only known mammalian iron exporter and is essential for the delivery of iron into plasma. Fpn is predominantly expressed on the basolateral side of enterocytes and on macrophages and hepatocytes (reviewed in Ref. [1]). The activity of Fpn is negatively regulated by the small peptide hormone hepcidin, which is released from the liver when there are sufficient levels of iron in circulation. Released hepcidin binds to the central cavity of Fpn and occludes it [2, 3], triggering endocytosis and proteolysis of the hepcidin‐Fpn complex, thus turning iron export off [4]. Dysregulation of the hepcidin‐Fpn axis is a principal driver of iron‐related disorders, including iron‐restricted anaemia in chronic inflammatory disorders (rheumatologic disorders, inflammatory bowel disease, cancers), infections and chronic kidney disease [5].

Given the role Fpn and hepcidin play in the normal cell and tissue function and in the pathophysiology of iron disorders, a detailed understanding of the Fpn transporter has outstanding biomedical significance. However, relatively little is known regarding Fpn structure, the molecular mechanism of Fpn‐mediated iron export and its regulation. In a recent breakthrough, the first structural insight into the Fpn transporter family was presented by the crystal structure of BbFpn, a prokaryotic Fpn orthologue from *Bdellovibrio bacteriovorous* [2]. The structure illustrated a major facilitator superfamily (MFS) type transporter fold, comprising of 12 transmembrane (TM) helices separated into N (TM1‐6)‐ and C‐terminal (TM7‐12) domains. A putative substrate site was identified in the structure, and modelling of the human protein based on the BbFpn structure suggested direct inhibition of transport by hepcidin, a conclusion recently supported by experimental evidence for the human Fpn protein [3].

Despite the valuable insights provided by the BbFpn structure, a number of questions relating to the structure and function of the Fpn transporters remain unanswered. To gain further insight, detailed biophysical studies of the mammalian protein are required. A prerequisite for this is to obtain sufficient quantities of stable and monodispersed protein in solution. However, this is often a significant hurdle for membrane proteins due to their low expression levels and poor stability. Approaches to increase the protein yield include codon optimization and changing constructs and/or expression system. Detergent screening can significantly increase protein stability [6, 7]. In addition, enhancing the intrinsic thermal stability of a protein through protein engineering, including loop deletions, N‐ and C‐terminal truncations and point mutations, is a common practice to obtain a sample conducible for biochemical and biophysical studies [8, 9, 10, 11].

Previously, Fpn from human, mouse and zebrafish was expressed using the baculoviral expression system and *Spodoptera frugiperda* (*Sf*9) cells [12]. At 50–100 μg Fpn per litre of insect cell culture, the expression level was relatively low, despite the use of an N‐terminal Rho tag which has been shown to increase the expression levels of eukaryotic membrane proteins [13]. Here, in an attempt to increase the yield of purified Fpn for downstream studies, we generated a fusion construct of a deglycosylation mutant of mouse Fpn and GFP for the baculoviral expression system. The purification of Fpn using our protocol produced ~ 1 mg of pure Fpn per litre of *Sf*9 cell culture, a more than 10‐fold increase from a previous protocol [12]. Furthermore, we generated a series of loop deletions that resulted in protein constructs with enhanced thermal stability when compared with that of the deglycosylation mutant. Transport activity and hepcidin responsiveness of the constructs were also analysed in the *Xenopus* oocyte expression system.

The development of a purification protocol yielding milligram quantities of pure Fpn and the generation of constructs with enhanced thermostability will facilitate studies aimed at illuminating Fpn structure and function. This can ultimately aid in our understanding of its role in the pathophysiology of iron disorders.

## Results and Discussion

### Topology and construct design

**Fig. 1:**
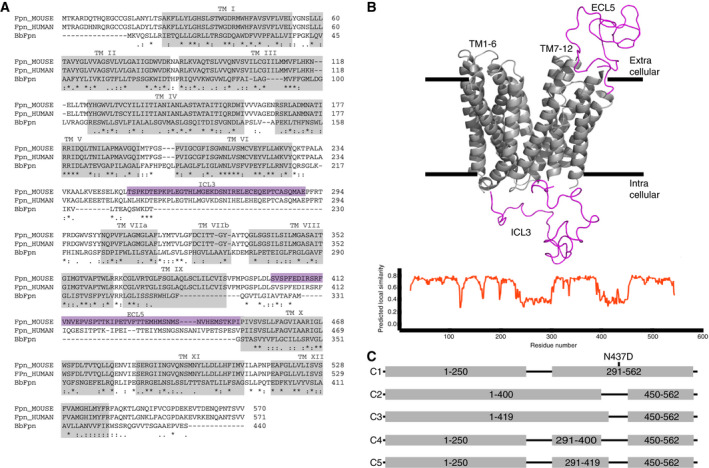
Topology and construct design. (A) Sequence alignment of human, mouse and *B. bacteriovorus* Fpn proteins. Fully conserved residues are indicated by a ‘*’, whilst strong and weak similarities are indicated by ‘:’ and ‘.’, respectively. The position of the TM segments from the structure of BbFpn and TOPCONS topology prediction is indicated with grey boxes. Larger loops present in the mammalian proteins, but not in the bacterial, are highlighted in purple. (B) Homology model of mouse Fpn generated using swiss‐model [30] (Univesity of Basel, Switzerland) and BbFpn as structural template (PDB code 5AYN). The ICL3 and ECL5 segments are shown in purple, and the predicted local similarity is illustrated (bottom). (C) Schematic of overall construct design. Numbers indicate residues included in each construct.

Predicting the topology for the mouse sequence, we obtained an 11 TM prediction using TOPCONS [14], which we adjusted to 12 TMs based on the structure of BbFPN and sequence alignment (Fig. 1). This adjusted topology is also largely in agreement with that of Liu *et al*. [15].

The mouse Fpn protein was previously shown to be subject to post‐translational modification by N‐linked glycosylation in HEK293 cell [16], although the exact residue(s) involved was not determined. By using the NetNGlyc server (www.cbs.dtu.dk/services/NetNGlyc/), we identified three predicted N‐linked glycosylation sites (N100, N174 and N437) in mouse Fpn. Considering the predicted topology, however, only N437 is expected to be extracellular and therefore the only residue that could be glycosylated. As glycosylation of proteins can result in less than optimal monodispersity of purified protein samples and impede biophysical studies, we generated an Fpn N437D mutant (Fpn^N437D^), which was used in subsequent studies and construct design.

Investigators often target loops for modification when seeking to increase the stability of a protein. We compared the sequences of mouse Fpn and the highly stable BbFpn and observed that the major observable differences between the two proteins are found in two loop regions, which are significantly longer in the mouse protein (Fig. 1A,B). The first loop, designated intracellular loop 3 (ICL3), connects the two 6TM domains of MFS transporters. The second loop, extracellular loop 5 (ECL5), connects TMs 9 and 10 through a ~ 50 residue long sequence. The function of this domain, if any, is unknown. It appears to be present only in vertebrates (data not shown), which may point to some role involving hepcidin binding.

We targeted these loops by generating deletion constructs that conceptually resemble (in overall topology) the BbFpn protein [2] (Fig. 1C). These constructs, designated C1 (ICL3 Δ251‐290), C2 (ECL5 Δ401‐449), C3 (ECL5 Δ420‐449), C4 (C1/C2) and C5 (C1/C3), were generated using overlapping PCR with a C‐terminal GFP‐His moiety with an intermittent TEV cleavage site. All five constructs and the N437D mutant on which they were based were sequence verified and transfected into *Sf*9 cells for viral propagation.

### Expression and purification of Fpn constructs

**Fig. 2:**
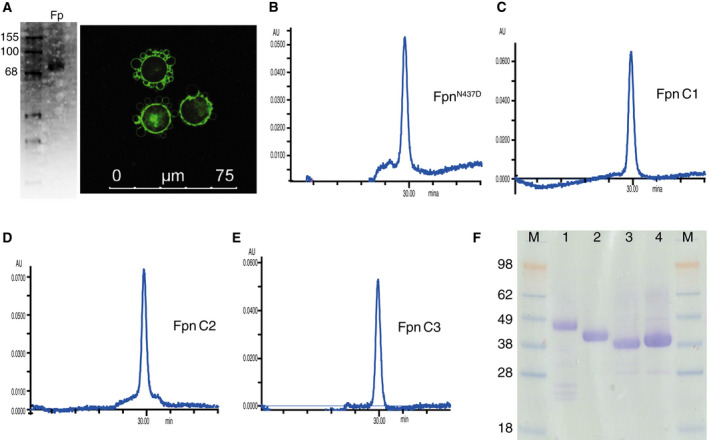
Purification of Fpn constructs. (A) Confocal microscopy image of *Sf*9 cells expressing Fpn^N437D^ –GFP and fluorescence image of SDS/PAGE analysis of whole cells. The Fpn^N437D^ –GFP fusion is detected at ~ 75 kDa. Length of the scale bar represents 75 μm (B) Analytical SEC profile of ~ 20 μg DDM purified Fpn^N437D^, (C) Fpn C1, (D) Fpn C4 and (E) Fpn C5. All SEC profiles indicate highly monodispersed sample. (F) SDS/PAGE analysis of purified protein constructs (1 – Fpn^N437D^; 2 – C1; 3 – C4; 4 – C5). Approximately 5 μg of purified protein were run under reducing conditions on a 4–12% Tris‐Glycine gel.

To obtain sufficient amounts of pure protein, we expressed the constructs in *Sf*9 cells infected with baculovirus at an optimal multiplicity of infection (MOI) (~ 2.5). In contrast to a previous study in which an N‐terminal Rho tag was required for detection [12], we observed high levels of membrane‐localized expression of Fpn^N437D^, C1, C4 and C5 constructs, as determined by GFP fluorescence (Fig. 2A).

To purify the highly expressing constructs, the protein samples were solubilized from the cell membrane using 1% *n*‐dodecyl‐β‐d‐maltoside (DDM), which has previously been shown to be effective in the solubilization of Fpn [12]. The protein was subsequently subjected to purification using Ni‐NTA and size exclusion chromatography (SEC), with the SEC profile of all constructs illustrating a highly monodispersed sample with near Gaussian distribution peak (Fig. 2B‐E). The eluted proteins were analysed on SDS/PAGE, which revealed Fpn^N437D^ to run with an apparent molecular mass of ~ 47 kDa, whilst the deletion constructs have apparent molecular masses of ~ 42, 38 and 40, respectively, for C1, C4 and C5 (Fig. 2F). Although lower than the anticipated molecular weight (Mw) of the constructs, membrane proteins commonly run at Mw significantly lower than their calculated Mw due to the intrinsic hydrophobic properties [17]. The final protein purity was estimated to be > 95% for all protein constructs based on the SDS/PAGE staining.

After the SEC, the protein was concentrated to ~ 10 mg·mL^−1^ and stored frozen until required. The overall yield of purified Fpn protein from each of these constructs was ~ 1 mg of protein per litre of cell culture.

### Assessment of Fpn thermal stability

The thermal stability of the purified proteins was assessed by monitoring the thermal melt curve using the thiol‐specific fluorochrome N‐[4‐(7‐diethylamino‐4‐methyl‐3‐coumarinyl)phenyl]maleimide (CPM) and slow (1 °C·min^−1^) temperature ramping in a qPCR. The CPM assay has been widely used to obtain a reproducible stability curve and melting temperature (*T*_m_, corresponding to the temperature where the protein is 50% unfolded) for proteins by monitoring the increased accessibility for CPM to buried cysteine residues as the protein unfolds [18]. The mouse Fpn sequence contains 13 cysteine residues, of which eight are located in predicted TM regions (four in the N‐terminal and four in the C‐terminal domains), whilst two are situated in ICL3 and are thus not present in the deletion constructs. The number and location of the TM‐embedded cysteine residues make the CPM assay suitable for the analysis of mouse Fpn stability.

**Fig. 3:**
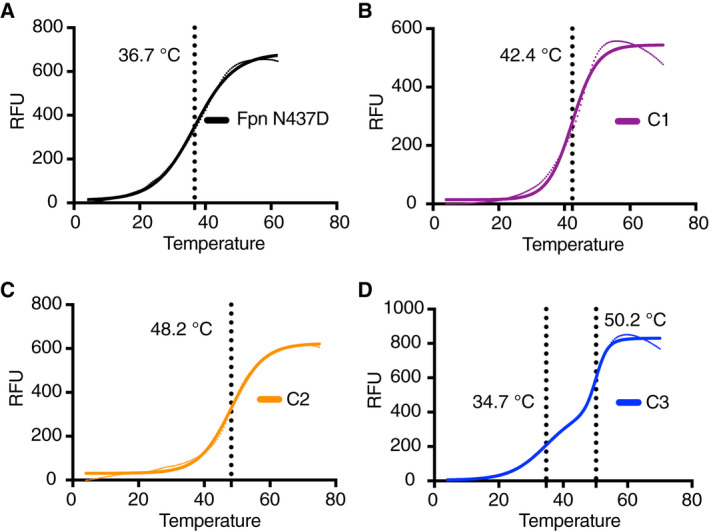
CPM stability assay of purified Fpn proteins. Representative thermal melt curves of Fpn^N437D^ (A), Fpn‐C1 (B), Fpn‐C4 (C) and Fpn‐C5 (D). The *T*_m_ of each construct is indicated by the vertical dotted line. The melt curve of Fpn^N437D^, Fpn‐C1 and Fpn‐C4 was best fit to a sigmoidal dose–response function, whilst the Fpn‐C5 construct was best fitted with a biphasic function.

The thermal melt curves of the purified Fpn proteins were analysed in Prism, and the apparent *T*_m_ of each construct was obtained by fitting the data by a sigmoidal dose–response curve. Under the experimental conditions, Fpn^N437D^ has a *T*_m_ of ~ 37 °C (Fig. 3A). By comparison, many membrane proteins used for biophysical and biochemical studies, with their thermal stability assessed by the CPM assay, have *T*_m_'s in the range of 40–60 °C [19, 20, 21, 22, 23]. This indicates that stability of Fpn^N437D^ is at the lower end of the spectrum for a protein conducible for *in vitro* studies. However, the *T*_m_ of the deletion constructs illustrate that these have an improved thermal stability; the apparent *T*_m_ of the C1 and C4 constructs are 44.0 and 46.7 °C (Fig. 3B,C), respectively. The enhanced thermostability of the C4 construct indicates that this construct has an increased structural order and reduced conformational flexibility, which may be favourable for biophysical studies, including structural studies where there is a strong correlation between thermal stability and potential to obtain highly ordered protein crystals [7, 18, 24]. Interestingly, the thermal melt curve of the C5 construct exhibited biphasic *T*_m_ (Fig. 3D), with $T_{m}^{1}$ of 35.3 °C and $T_{m}^{2}$ of 50.5 °C. As such, it is possible that the partial deletion of ICL5 has caused local instability, possibly in the C‐terminal 6TM domain, leading to a hierarchical unfolding of the protein.

### Transport activity and hepcidin responsiveness in the *Xenopus* oocyte expression system

**Fig. 4:**
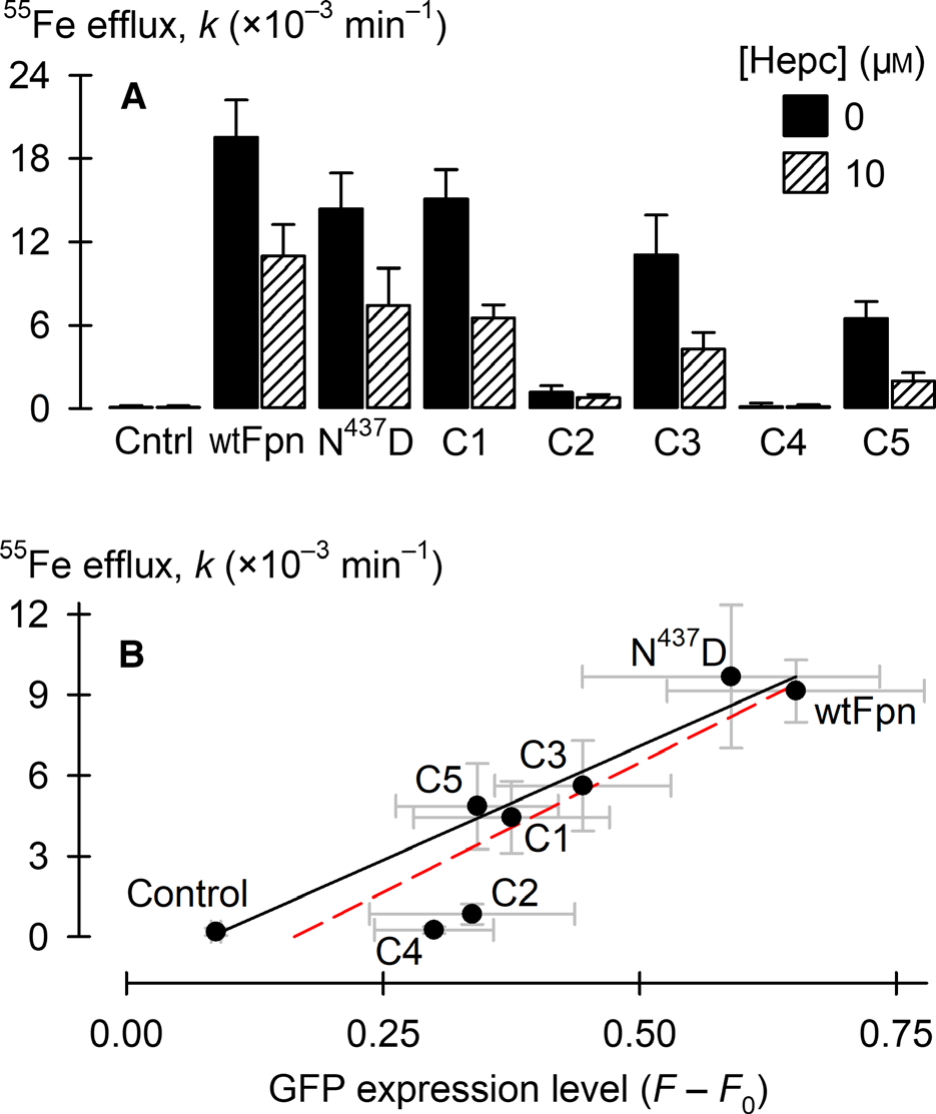
Activity and hepcidin sensitivity of mouse Fpn constructs expressed in RNA‐injected *Xenopus* oocytes. (A) ^55^Fe efflux activity (*k*, first‐order rate constant) in control oocytes (Cntrl) and oocytes expressing mouse Fpn constructs, pretreated for 30 min in the absence or presence of 10 µm hepcidin (Hepc). *n* = 9–12 oocytes per group. Two‐way ANOVA: interaction, *P* < 0.001. Within 0 μm Hepc, all constructs differed from control (*P* < 0.001) except C2 (*P* = 0.26) and C4 (*P* = 0.92). Within individual Fpn constructs, 10 μm Hepc differed from 0 (*P* < 0.001) except in the cases of C2 (*P* = 0.60) and C4 (*P* = 0.99). (B) ^55^Fe efflux activity (*n* = 10–18 oocytes per group) as a function of protein expression as measured by GFP fluorescence (*n* = 4–5 oocytes per group) in the same oocyte preparation. Data for all constructs and control oocytes were fit by a linear function (red dashed line, adjusted *r* ^2^ = 0.78, *P* < 0.001). When C2 and C4 were removed from the regression (black solid line, adjusted *r* ^2^ = 0.95, *P* < 0.001), we obtained an improved fit (*P* = 0.005 in an *F* test of the model improvement, by using the sum of the squares of the residuals). Error bars represent SD.

We analysed the transport activity and hepcidin responsiveness of the deletion constructs by using a radiotracer assay in RNA‐injected *Xenopus* oocytes expressing mouse Fpn constructs. Wild‐type mouse Fpn, Fpn^N437D^ and the C1, C3 and C5 constructs exhibited ^55^Fe efflux activity, whereas the C2 and C4 constructs exhibited no discernible activity (Fig. 4A). This result was replicated in two additional oocyte preparations. Hepcidin pretreatment (10 µm, 30 min) inhibited the ^55^Fe efflux activity of each of the Fpn constructs for which we observed transport activity. In an independent oocyte preparation, we related ^55^Fe efflux activity to the expression levels of GFP fusion protein by using confocal microscopy. We found that differences in absolute ^55^Fe efflux activity were generally explained by the variation in expression levels, except in the cases of the C2 and C4 constructs (Fig. 4B). Despite their detectable expression, C2 and C4 lacked iron‐transport activity. Therefore, loss of residues 400–419 disrupts iron‐transport activity of mouse Fpn expressed in oocytes.

## Conclusions

We have generated a robust expression and purification protocol for mouse Fpn, yielding pure and monodisperse protein. Our new protocol provides for a substantially improved yield compared with the protocol previously described for Fpn using the same expression system [12]. In addition, we have made selective deletion constructs that exhibit improved thermal stability in solution and largely retain iron‐transport activity *in vitro*. During the review process of this manuscript, the structure of human Fpn was published [25]. Whilst this provides an important advancement, the detailed transport mechanism remains unresolved. As such, our protocol may constitute a platform for future structural and biophysical studies.

## Materials and methods

### Materials

Oligonucleotides were from IDT DNA (San Diego, CA, USA). Phusion High‐Fidelity DNA polymerase, enzymes for DNA manipulation and endoglycosidases were from New England Biolabs (Beverly, MA, USA). All PCR and cloning steps were performed using established protocols [26]. Bac‐to‐Bac Baculovirus Expression System and antibodies were purchased from Thermo Fisher Scientific (Waltham, MA, USA). Insect‐XPRESS^™^ Protein‐free Insect Cell Medium with l‐glutamine was from Lonza (Basel, Switzerland). Ni‐NTA resin was purchased from Qiagen (Valencia, CA, USA), and polyprep columns were from Bio‐Rad (Hercules, CA, USA). All other reagents were purchased from Sigma‐Aldrich (St. Louis, MO, USA), unless otherwise noted.

### Topology prediction

The topology of mouse Fpn was based on a combination of (a) the TOPCONS server [14], which in turn combines the topology prediction from five ‘submethods’ (OCTOPUS, Philius, PolyPhobius, SCAMPI and SPOCTOPUS) into a consensus topology profile; using (b) sequence alignment with BbFpn using clustal omega [27]; and (c) the structure of BbFpn (PDB entry code 5AYN).

### Fpn gene cloning and protein expression

All constructs generated in this study were engineered to have the gene sequence followed by a TEV cleavage site, GFP and an 8xHis tag. The constructs and the respective primers are listed in Table 1. The wild‐type Fpn construct was generated from full‐length mouse Fpn (UniProt: Q9JHI9). The gene sequence was fused with a C‐terminal TEV‐GFP‐8xHis sequence amplified from the GFP fusion vector pDDGFP‐2 [28] and the fusion construct cloned into a NotI/XhoI‐digested baculovirus transfer vector (pFastBac1; Thermo Fisher Scientific) and sequence verified. The potential N glycosylation site (N437) was mutated to an Asp residue to generate Fpn‐N437D (Fpn^N437D^) construct. Briefly, the forward and reverse primers were designed to incorporate nucleotide change of the Asn to Asp residue located in the middle of the primer. Two PCR fragments were generated for regions comprising residues 1‐437 and 437‐end of construct comprising the 8xHis tag. The fragments were joined into one reading frame by overlap extension PCR using primer pairs WT‐Fpn‐F and R‐His. To obtain a stable mammalian Fpn protein conducive for biophysical studies, we generated and tested five loop deletion constructs using Fpn^N437D^ as a template. A similar strategy of overlap PCR was used for cloning where the primers were designed to contain the gene sequence flanking the desired deletion, and the 3′ end of the reverse primer was designed complementary to the 5′ end of the forward primer sequence. Construct ‘Fpn‐C1’ was used as the template to generate constructs ‘Fpn‐C4’ and ‘Fpn‐C5’. All constructs were cloned into a NotI/XhoI‐digested pFastBac1vector. The baculoviral stock production (P1‐P3) and amplification for each construct was performed using the Bac‐to‐Bac Baculovirus Expression method according to the manufacturer's protocol (Thermo Fisher Scientific). For large scale expression of the protein, 6L *Sf*9 cells (2 × 10^6^ cells per mL) were infected with P3 virus at an optimal MOI of 2.5 in Insect‐XPRESS^™^ Protein‐free Insect Cell Medium with l‐glutamine (Lonza) and incubated at 27 °C with shaking at 130 r.p.m. The cells were spun down 48 h postinfection at 1200 ***g*** for 10 min, resuspended in 20 mm Tris pH 8, 300 mm NaCl (Buffer A) and stored at −20 °C until further use.

**Table 1:** Primer sequences.

Construct name	Description	Primer name	Primer sequence
wt‐Fpn	WT Mouse FPN	WT‐FPN‐F	TCTTTTTGCGGCCGCGCCACCATGACCAAGGCAAGAGATC
		WT‐FPN‐R	CCT TGA AAA TAT AAA TTT TCC CCA GAT CC TAC AAC AGA TGT ATT CGG TTG ATT TTC
		R‐His	AGA AAT CTA GAC TCG AGT TAA TGA TGA TGA TGA TGG TGG
Fpn^N437D^	Single glycosylation mutant	N437D‐F	ACAGAAATGCATATGTCCGACATGTCT
		N437D‐R	CATGGACATTAGACATGTCGGACATAT
Deletion constructs			
Fpn‐C1	Δ251‐290	C1‐F	AAGCAGCTGACCCCCTTCCGCACTTTCCGAGA
		C1‐R	AGTGCGGAAGGGGGTCAGCTGCTTCAGTTCTG
Fpn‐C2	Δ400‐450	C2‐F	CCCTTGGACCTGCCCATAGTCTCTGTCAGCCTGC
		C2‐R	AGAGACTATGGGCAGGTCCAAGGGGCTTCCAG
Fpn‐C3	Δ420‐450	C3‐F	CCAGTGTCCCCACCCATAGTCTCTGTCAGCCTGC
		C3‐R	AGAGACTATGGGTGGGGACACTGGCTCCACAT

### Fpn protein purification

For the purification of all Fpn constructs, the *Sf*9 cells were thawed at room temperature with the addition of 2 μL Benzonase (250 units per μL), 1 mm MgSO_4_ and 0.2 mm PMSF and lysed by a single pass through cooled EmulsiFlex‐C3 homogenizer (Avestin, Ottawa, Canada). All subsequent steps were performed at 4 °C. Membranes were harvested by centrifugation at 106 000 ***g*** for 1 h, and pellets resuspended in Buffer A.

Membrane proteins were solubilized by the addition of 1% (final concentration) DDM and incubated under stirring for 1 h. Insoluble cellular debris was subsequently cleared by centrifugation at 106 000 ***g*** for 20 min. The supernatant containing the solubilized protein was incubated with Ni‐NTA (Qiagen) and pre‐equilibrated with Buffer A, for 2 h under slow stirring. The protein‐bound resin was loaded onto a polyprep gravity flow column (Bio‐Rad), and the resin was subsequently washed with (a) 20 column volumes of 20 mm imidazole pH 8, 300 mm NaCl and 0.03% DDM (Buffer B) and (b) 3 column volumes of 25 mm imidazole pH 8, 300 mm NaCl and 0.03% DDM. Protein was then eluted with buffer containing 200 mm imidazole, 300 mm NaCl and 0.03 % DDM. The GFP moiety was cleaved from the eluted protein using TEV protease under dialysis with Buffer A NaCl (overnight). The GFP moiety and TEV protease were cleared from Fpn by rebinding dialysed protein to Ni‐NTA. The untagged protein was eluted and concentrated to 1.8 mL before loading onto a Superdex 200 16/600 size exclusion column (GE Healthcare Life Sciences, Logan, UT, USA) and pre‐equilibrated with 20 mm Tris pH 8, 50 mm NaCl and 0.03 % DDM (SEC buffer). The eluted protein was concentrated to ~ 10 mg·mL^−1^ as determined by the BCA assay (Thermo Fisher Scientific) method and stored at −80 °C until further use.

### Thermal stability assay

The thermal stability of Fpn proteins was evaluated using the thiol‐specific fluorochrome CPM, as described previously [18], with minor modifications. Briefly, 1.2 μL of purified Fpn protein (~ 0.2 nmol) was diluted to 18 μL with 20 mm Tris pH 6.5, 50 mm NaCl and 0.03% DDM and subsequently mixed with 0.5 μL CPM dye (4 mg·mL^−1^, final concentration 0.1 mm). The mixture was incubated on ice for 10 min and subsequently transferred to a clean PCR tube and heated with a ramp rate of 1 °C·min^−1^ in a My iQ2 thermal cycler (Bio‐Rad). The excitation wavelength was set to 365 nm, and the emission wavelength was set to 460 nm. Assays were performed over a temperature range of 4–80 °C. Each experiment was performed at least two times.

### Functional expression of mouse Fpn constructs in *Xenopus* oocytes

We performed laparotomy and ovariectomy on adult female *Xenopus laevis* frogs (Nasco, Fort Atkinson, WI, USA) under 2‐aminoethylbenzoate methanesulfonate anaesthesia (0.2%, by immersion, to effect) following a protocol approved by the University of Cincinnati Institutional Animal Care and Use Committee. Oocytes were prepared and stored as described [29]. Wild‐type mouse Fpn cDNA (C. R. Azucenas, T. A. Ruwe, J. P. Bonamer, B. Qiao, T. Ganz, M. Jormakka, E. Nemeth, & B. Mackenzie, unpublished data), N437D, and constructs C1–C5, each tagged at the C terminus with GFP, were subcloned into the pOX(+) oocyte expression vector. Oocytes were injected with ~ 50 ng of RNA and incubated 4–5 days before being used in functional assays. ‘Control’ oocytes were noninjected. We determined GFP fusion protein expression levels by using confocal laser scanning microscopy and measured iron efflux from oocytes by using a radiotracer assay, both as described [1, 3, 29] and expressed activity as the first‐order efflux rate constant (*k*). Where noted, oocytes were pretreated 30 min with 10 µm human hepcidin (Peptides International).

### Statistical analysis

Data are expressed as mean, SD for *n* independent observations. We set critical significance level α = 0.01 (oocyte assays) and analysed our data by using sigmaplot v14 (Systat Software, San Jose, CA, USA). We used two‐way analysis of variance (ANOVA) followed, when appropriate, the Holm–Šidák test for multiple pairwise comparisons and linear regression analysis. *T*_m_ were determined by fitting the data by a sigmoidal dose–response, or biphasic, curve using graphpad prism software (v4.0; GraphPad Software Inc., San Diego, CA, USA). Log EC50 calculations were performed using the built‐in algorithms.

## Conflict of interest

The authors declare competing financial interests: TG and EN are scientific advisers to and shareholders in Intrinsic LifeSciences and Silarus Therapeutics and consultants for La Jolla Pharmaceutical Company and Keryx Pharmaceuticals. BM is a grant recipient of Vifor Pharma.

## Author contributions

EN and MJ conceptualized the study. CND, CRA, BQ, NN, VX, JF, BM, TG, SI, EN and MJ designed and performed experiments and analysed the data. CND, NN, EN, BM and MJ wrote the manuscript. All authors edited the manuscript and approved the final version.

## Data Availability

Data are available from the corresponding author upon reasonable request. Correspondence and requests for materials should be addressed to MJ (m.jormakka@centenary.org.au).
